# Supplementary material for: Bayesian polygenic risk estimation approach to nuclear families with discordant sib-pairs for myelomeningocele
Source: PLoS One. 2024 Dec 30;19(12):e0316378. doi: 10.1371/journal.pone.0316378 (PMC11684611; doi:10.1371/journal.pone.0316378)
Supplement: S1 File — (DOCX) [file pone.0316378.s003.docx]

**Bayesian Analysis**

**Model Specification**

We conducted a Bayesian analysis based on Bayes' theorem to estimate the MMC risk associated with allele transmission. This approach combines prior knowledge of MMC prevalence with observed allele frequencies to compute the posterior probability of developing the disease, given exposure to specific genetic variants.

**Bayes' Theorem Framework:**

$$P\left( \text{Disease} \mid\text{Exposure} \right)=\frac{P\left( \text{Exposure} \mid\text{Disease} \right)\times P\left( \text{Disease} \right)}{P\left( \text{Exposure} \right)}$$

Where:

$P\left( \text{Disease} \mid\text{Exposure} \right)$: Posterior probability of developing MMC given exposure to the risk allele.

$P\left( \text{Exposure} \mid\text{Disease} \right)$: Likelihood of observing the risk allele in cases.

$P\left( \text{Disease} \right)$: Prior probability of MMC (prevalence).

$P\left( \text{Exposure} \right)$: Marginal probability of exposure to the risk allele.

**Parameters and Priors**

Transmitted and not transmitted risk alleles frequencies in cases and siblings from TDT-DS [1] (Table 3):

$f_{\text{MMC-sibs}}$: Frequency of the transmitted risk alleles in MMC-affected sibs.

$f_{\text{Non-affected-sibs}}$: Frequency of the transmitted risk alleles in Non-affected sibs.

MMC Prevalence ($P\left( \text{Disease} \right)$):

Pre-FAFP (Before Folic Acid Fortification Program): 1.098 per 1,000 births (or 1/910).

Post-FAFP (After Folic Acid Fortification Program): 0.562 per 1,000 births (or 1/1,779).

Data sourced from RYVEMCE registry spanning 1978–2002 (pre-FAFP) and 2003–2019 (post-FAFP) [2].

**Posterior Probability Calculations**

**For Individual Variants:**

The posterior probability of disease given exposure to a single variant was calculated as:

$$P\left( \text{MMC} \mid\text{Exposed to Variant }i \right)=\frac{f_{\text{MMC-sibs}}^{\left( i \right)}\times P\left( \text{MMC} \right)}{f_{\text{MMC-sibs}}^{\left( i \right)}\times P\left( \text{MMC} \right)+f_{\text{Non-affected-sibs}}^{\left( i \right)}\times\left( 1-P\left( \text{MMC} \right) \right)}$$

Where:

$f_{\text{MMC-sibs}}^{\left( i \right)}$ frequency of the TDT transmitted alleles in MMC-affected sibs.

$f_{\text{Non-affected-sibs}}^{\left( i \right)}$frequency of the TDT transmitted alleles in Non-affected sibs.

$P\left( \text{MMC} \right)$prevalence of MMC.

$1-P\left( \text{MMC} \right)$ probability of not having MMC.

**For Combined Variants:**

Assuming independent effects, the combined posterior probability for multiple variants is calculated as follows:

$$P\left( \text{MMC} \mid\text{Exposed to Variants }i,j,\ldots\right)=\frac{\prod_{k} f_{\text{MMC-sibs}}^{\left( k \right)}\times P\left( \text{MMC} \right)}{\prod_{k} f_{\text{MMC-sibs}}^{\left( k \right)}\times P\left( \text{MMC} \right)+\prod_{k} f_{\text{Non-affected-sibs}}^{\left( k \right)}\times\left( 1-P\left( \text{MMC} \right) \right)}$$

Where:

$k$: indexes each variant in the combination.

$f_{\text{MMC-sibs}}^{\left( k \right)}$​: frequency of the transmitted alleles for variant $k$ in MMC-affected sibs.

$f_{\text{Non-affected-sibs}}^{\left( k \right)}$: frequency of the transmitted alleles for variant $k$ in Non-affected sibs.

$P\left( \text{MMC} \right)$: prevalence of MMC in the population.

**Assumption of Independent Effects**

We assumed that the genetic effects of multiple variants are independent when combined. While this simplifies the calculations for polygenic risk estimation, it does not account for interactions between variants. Although this assumption is practical for estimating genetic risk, we acknowledge that certain variants may biologically interact, which could affect the accuracy of risk estimates. Independence is a reasonable assumption when variants are located in different genes or biological pathways with no known interactions. However, if variants are in linkage disequilibrium or involved in the same biological pathway, this assumption may not hold, potentially leading to biased estimates. To mitigate this, we selected only one variant per gene to minimize multicollinearity and linkage effects.

**Implementation Using Shiny Application**

A Shiny application was developed in R to perform Bayesian probability calculations, providing users with an interactive and dynamic interface. The application offers several features. Users can upload a CSV file with allele frequencies in family quartets. It supports the analysis of multiple genetic variants, enabling individual and combined analyses. Additionally, users can choose to calculate probabilities such as $P\left( \text{MMC} | \text{Exposed} \right)$, $P\left( \text{MMC} | \text{Non-Exposed} \right)$, or both. The results are displayed directly within the application, with an option to download the output in CSV or TXT format for further use. The complete code is provided as a supplementary file (bayes_calculator.R).

**Variant Categorization and Prioritization**

A systematic approach was implemented to variant categorization and prioritization. Variants were classified into two main categories: missense and non-missense variants (intron and synonymous). Missense variants refer to changes in nucleotide sequences that lead to amino acid substitutions, potentially altering protein function. Non-missense variants include synonymous variants, which do not result in amino acid changes, and intronic variants, which occur in non-coding regions but may influence gene expression regulation. Ensembl Variant Effect Predictor (VEP) was used as our primary annotation tool to provide comprehensive predictions on the impact of each variant. We selected only one variant per gene when multiple variants were identified within the same gene. The chosen variant was either the one with the highest predicted functional impact or the most significantly associated with MMC. This approach helped avoid linkage effects by minimizing the inclusion of additional variants from the same gene, reducing the potential confounding due to linkage disequilibrium.

**References:**

1. Deng H-W, Chen W-M, Recker RR. Transmission disequilibrium test with discordant sib pairs when parents are available. Hum Genet. 2002;110: 451–461. doi:10.1007/s00439-002-0675-9.

2. Mutchinick O, Lisker R, Babinski V. Programa Mexicano de “Registro y vigilancia epidemiológica de malformaciones congénitas.” Salud Pública México. 1988;30: 88–100. Available: https://www.saludpublica.mx/index.php/spm/article/view/214

**Supplementary Files:**

- Shiny Application Code: Full R script for the Bayesian probability calculator ("bayes_calculator.R").

- Data Files: Example CSV files for input into the Shiny application ("bayes_6varsp05.csv").
