# Supplementary material for: Bayesian polygenic risk estimation approach to nuclear families with discordant sib-pairs for myelomeningocele
Source: PLoS One. 2024 Dec 30;19(12):e0316378. doi: 10.1371/journal.pone.0316378 (PMC11684611; doi:10.1371/journal.pone.0316378)
Supplement: Attachment — Submitted filename: PLos2-BayesianPolygenicRisk_Review.pdf [file pone.0316378.s006.pdf]

Review of "Bayesian Polygenic Risk Estimation Approach to Nuclear Families with Discordant Sib-Pairs for Myelomeningocele"

May, 2024

In this paper, the authors have adopted a Bayesian approach to estimate polygenic risk and identify the significant genes related to neural tube disorders (NTDs). While the topic of the paper is significant, the paper should be better constructed and include more details about the tests, mechanisms used, assumptions, modeling techniques etc. For example:

1. Section 2.3.3: Bayesian Analysis: Bayesian is an estimation approach which allows one to incorporate prior knowledge about model parameters. It performs estimation through posterior sampling. However, it still needs a particular model to work on. The stats section in the paper lacks proper description of the exact model that was used, the model parameters, what prior were assumed in the Bayesian analysis and how posterior sampling was done.

2. Line 134: "The analysis were conducted by categorizing gene variants into missense and non-missense..." - How was this categorization done?

3. "Independent effects are assumed when combining the effects of multiple variants" - can the authors discuss the practicality of this assumption and what happens when it is violated.

4. Bayesian Analysis Results: Can the authors provide some measures of model diagnostics and time complexity?

   General comment: The authors should include more details and be specific about the analysis performed for completion.
