# Supplementary material for: Bayesian polygenic risk estimation approach to nuclear families with discordant sib-pairs for myelomeningocele
Source: PLoS One. 2024 Dec 30;19(12):e0316378. doi: 10.1371/journal.pone.0316378 (PMC11684611; doi:10.1371/journal.pone.0316378)
Supplement: Attachment — Submitted filename: Response to Reviewers.docx [file pone.0316378.s007.docx]

**October 21, 2024**

**Rebuttal letter**

Dear Editor-in-Chief,

Dear Academic Editor and Reviewers,

We sincerely appreciate all reviewers' insightful feedback, which allowed us to significantly improve the clarity and depth of our manuscript, **"Bayesian Polygenic Risk Estimation Approach to Nuclear Families with Discordant Sib-pairs for Myelomeningocele."**

In this rebuttal letter, we have carefully addressed all the reviewers' comments and made the corresponding revisions to the manuscript. Specifically, we have clarified the Bayesian framework used, expanded the methodological details, and provided further discussion on the assumptions and computational aspects of the analysis.

We hope the revised manuscript answers the reviewers' comments and suggestions.

Sincerely yours,

**Osvaldo M. Mutchinick, MD, PhD**

Professor of Medical Genetics

Chief, Department of Genetics

National Institute of Medical Sciences and Nutrition Salvador Zubirán

Vasco de Quiroga 15, Col. Belisario Domínguez, Tlalpan

14080, Mexico City, Mexico

Phone: (5255) 5487-0900, Ext. 2514/2515

Direct Phone: (5255) 5655-6138

E-mail: [osvaldo@unam.mx](mailto:osvaldo@unam.mx)

**Response to Journal Requirements**

We have carefully reviewed the journal's additional requirements and have addressed each of them as follows:

1. *PLOS ONE Style Requirements*

We have ensured that the revised manuscript meets PLOS ONE's style requirements.

2. *Data Availability Statement*

We confirm that our submission includes all the raw data required to replicate our study's results. These data are included within the manuscript and its "supporting Information files" to ensure full reproducibility. Should the reviewers or editors require additional data, we agree to provide them upon request.

3. *Ethics Statement.*

We have included a complete ethics statement in the 'Methods' section of the revised manuscript. This statement specifies the name of the Institutional Review Board (IRB) or Ethics Committee that approved the study, the written consent procedures, and any relevant waivers obtained. We hope these revisions meet the journal's requirements, and we remain available for any further clarifications.

**Response to reviewers**

**Reviewer #1:**

**Observation 1**. Section 2.3.3: Bayesian Analysis: Bayesian is an estimation approach which allows one to incorporate prior knowledge about model parameters. It performs estimation through posterior sampling. However, it still needs a particular model to work on. The stats section in the paper lacks proper description of the exact model that was used, the model parameters, what prior were assumed in the Bayesian analysis and how posterior sampling was done.

**Response:**

We have removed the section numbering from the document. The "Bayesian analysis" section, previously identified as 2.3.3, starts on line 126 of the revised manuscript.

We would like to clarify that our approach employs Bayesian principles to estimate the probability of neural tube defects (NTDs) based on allele transmission frequencies. However, it does not involve a formal Bayesian regression model or posterior sampling. Instead, we use a Bayesian probabilistic framework to adjust for known MMC prevalence, which acts as our prior and calculates posterior probabilities of disease given exposure to specific genetic variants.

Our model framework is based on Bayes' Theorem, where the prior probability is the population prevalence of MMC, and the likelihood is based on the observed allele frequencies in both MMC-affected and non-affected sibs. Specifically, the posterior probability of disease given exposure to a genetic variant is estimated using the following formula:

$$P\left( \text{MMC} | \text{Exposed to}\text{ TDT}\text{ }\text{transmitted }\text{Variant }\text{i} \right)=\frac{f_{\text{MMC-sibs}}^{\left( i \right)}\times P\left( \text{MMC} \right)}{f_{\text{MMC-sibs}}^{\left( i \right)}\times P\left( \text{MMC} \right)+f_{\text{Non-affected-sibs}}^{\left( i \right)}\times\left( 1-P\left( \text{MMC} \right) \right)}$$

Where:

- $f_{\text{MMC-sibs}}^{\left( i \right)}$: Is the frequency of the TDT-transmitted allele in MMC-affected sibs.

- $f_{\text{Non-affected-sibs}}^{\left( i \right)}$ : Is the frequency of the TDT transmitted allele in Non-affected sibs.

- $P\left( \text{MMC} \right) :$ Is the prevalence of MMC.

**Observation 2**. Line 134: "The analysis were conducted by categorizing gene variants into missense and non-missense..." - How was this categorization done?

**Response:**

We classified variants into two main categories: missense variants and non-missense variants. Missense variants are nucleotide changes that lead to amino acid substitutions, potentially altering protein function. Non-missense variants include synonymous variants, which do not change the amino acid sequence, and intronic variants, which occur in non-coding regions but may affect gene expression regulation. We used the Ensembl Variant Effect Predictor (VEP) for variant annotation. VEP provided comprehensive predictions on the functional impact of each variant, which we used for categorization. When multiple variants were identified within the same gene, we selected the variant with the highest predicted functional impact or most significantly associated with MMC. More detailed descriptions of the categorization process are provided in the supplementary file.

**Observation 3.** "Independent effects are assumed when combining the effects of multiple variants" - can the authors discuss the practicality of this assumption and what happens when it is violated.

**Response:**

The assumption of independent effects simplifies the calculation of polygenic risk by allowing us to combine the effects of multiple variants multiplicatively, as follows:

$$P\left( \text{MMC} | \text{Exposed to variants }\text{i,j,}\text{…} \right)=\frac{\prod_{k} f_{\text{MMC-sibs}}^{\left( k \right)}\times P\left( \text{MMC} \right)}{\prod_{k} f_{\text{MMC-sibs}}^{\left( k \right)}\times P\left( \text{MMC} \right)+\prod_{k} f_{\text{Non-affected-sibs}}^{\left( k \right)}\times\left( 1-P\left( \text{MMC} \right) \right)}$$

This assumption holds when the variants are located in different genes or pathways and do not interact biologically. The above is reasonable for variants that are unlinked and functionally independent. However, when variants are in linkage disequilibrium or are part of the same biological pathway, this assumption may be violated, leading to biased estimates. In such cases, interactions between variants could amplify or suppress the individual effects, which our method does not capture. To mitigate this issue, we selected one variant per gene to minimize potential linkage effects and reduce multicollinearity, thus limiting the risk of confounding due to interactions between linked variants. We recognize that this approach may miss some gene-gene interactions and discuss this limitation.

**Observation 4.** Bayesian Analysis Results: Can the authors provide some measures of model diagnostics and time complexity?

**Response:**

Traditional convergence diagnostics are not applicable since we did not employ a formal Bayesian regression model with posterior sampling. Our approach involves direct calculation of posterior probabilities rather than iterative sampling. However, we can discuss the computational complexity of the analysis. The complexity is driven by the combinatorial nature of calculating probabilities for multiple variants. Given (*n*) variants, the number of combinations we need to evaluate is ($2^{n}-1$). For each combination, we compute the posterior probabilities. Time complexity grows exponentially with the number of variants, but we limit the number of variants per analysis for practical purposes to maintain computational feasibility. We also employed progressive computation in our Shiny application to provide real-time feedback on the calculations, improving user experience.

**General comment:** The authors should include more details and be specific about the analysis performed for completion.

**Response:**

We appreciate the feedback and revised the manuscript to include more details about the analysis, especially about the categorization and selection of variants, the probabilistic framework used for Bayesian analysis, and the assumptions of independence. Additionally, we will provide more specific information about the computational aspects of our methodology, including the limitations of the independent effects assumption and the steps we took to mitigate its potential violations. We hope that our responses clarify and address all comments, and we are open to further enriching our manuscript based on any additional suggestions.

**Reviewer #2 and Reviewer #3:** The authors sincerely appreciate positive comments on the study. We also thank all the reviewers for their time and thoughtful review of our manuscript.
