# Supplementary material for: Bayesian polygenic risk estimation approach to nuclear families with discordant sib-pairs for myelomeningocele
Source: PLoS One. 2024 Dec 30;19(12):e0316378. doi: 10.1371/journal.pone.0316378 (PMC11684611; doi:10.1371/journal.pone.0316378)
Supplement: Attachment — Submitted filename: Response to Reviewers.docx [file pone.0316378.s008.docx]

**Response to Journal Requirements**

We have carefully reviewed the journal's additional requirements and have addressed each of them as follows:

1. *Completeness and correctness of the reference list:*

- We realized that references number 4 and 40 were duplicates. Reference 40 (Lines 416-418) was eliminated, and the annotation of the other references was corrected in the text (lines 246, 266, 273, and 299) and the final list of references (lines 419, 421, 425, 429, and 432).
- Reference 5 (lines 315-318) was corrected to meet the journal's guidelines.
- Reference 35 (lines 401-403): the DOI was added, and the hyperlink to the article was eliminated.

2. *Revision of the cited articles:*

- We reviewed all cited articles by searching in the *Retraction Watch Database* [1], *CrossRef* [2], *Google Scholar* [3], *PubMed* [4], and directly on the websites of the journals where the articles were published. None of the 44 references appeared retracted.

[1]. Retraction Watch. Retraction Watch Database [Internet]. New York: The Center for Scientific Integrity; c2024 [cited 2024 Nov 26]. Available from: <https://retractionwatch.com/the-retraction-watch-database/>

[2]. CrossRef. CrossRef Metadata Search [Internet]. Lynnfield: CrossRef; c2024 [cited 2024 Nov 26]. Available from: <https://search.crossref.org/>

[3] Google Scholar. Google Scholar [Internet]. Mountain View: Google; c2024 [cited 2024 Nov 26]. Available from: <https://scholar.google.com/>

[4] National Center for Biotechnology Information (NCBI). PubMed [Internet]. Bethesda: U.S. National Library of Medicine; c2024 [cited 2024 Nov 26]. Available from: <https://pubmed.ncbi.nlm.nih.gov/>

**Response to reviewers**

**Reviewer #2:**

**Comment 6**. "*This revision addressed most previous comments, and the research was presented in a clear and informative manner. Some figures and tables, however, need to be adjusted in size for better readability (e.g. Line 163-164, the last column of Table 1 showing HGVSp extends out of the page; line 223, figure 1 is not there). Other issues mostly involve small grammar mistakes (e.g. Line 173, a period punctuation mark is missing) and long sentences that are generally acceptable*."

**Response:**

- Regarding Table 1, we retained its original size as it was necessary to ensure clarity and readability, modifying the paper sheet size.
- Regarding Figure 1, the figure was not placed in the main manuscript, as stipulated in the journal's guidelines (https://journals.plos.org/plosone/s/submission-guidelines#loc-references):

"Do not include figures in the main manuscript file. Each figure must be prepared and submitted as an individual file. … Figure captions are inserted immediately after the first paragraph in which the figure is cited. Figure files are uploaded separately..."

- To ensure compliance, we checked the figure using the PACE tool (https://pacev2.apexcovantage.com/). The adjustments were as follows: the image file was flattened, dimensions were adjusted to 7.49 in W x 4.69 in H, and the TIF file was converted to a valid TIF format. Following these changes, we uploaded a new figure file.
- We have thoroughly reviewed the manuscript for grammatical errors and addressed several issues, including:
  - Line 81: The word "off" was deleted
  - Line 173: Final punctuation corrected.
  - Line 181: Extra space removed before the number "0.001".
  - Lines 118, 186, 195, 198, 212, 227, 244, 266, 433: "TDTDS" changed to "TDT-DS" for better clarity.
  - S1 File and S2 Table: "TDTDS" changed to "TDT-DS"
  - Lines 211, 238-241, 250-252, 258, 267, 268, 271, 273, 274: Gene names formatted in italics.
  - Line 233: Space added after the word "model."
  - Line 255: "increased" changed to "increase."
  - Line 281: "pre" changed to "pre-."
  - Line 296: "ad hoc" formatted in italics.
